# A deep learning model to generate synthetic CT for prostate MR-only radiotherapy dose planning: a multicenter study

**DOI:** 10.3389/fonc.2023.1279750

**Published:** 2023-11-28

**Authors:** Safaa Tahri, Blanche Texier, Jean-Claude Nunes, Cédric Hemon, Pauline Lekieffre, Emma Collot, Hilda Chourak, Jennifer Le Guevelou, Peter Greer, Jason Dowling, Oscar Acosta, Igor Bessieres, Louis Marage, Adrien Boue-Rafle, Renaud De Crevoisier, Caroline Lafond, Anaïs Barateau

**Affiliations:** ^1^ University of Rennes, Centre de Lutte contre le Cancer (CLCC) Eugène Marquis, INSERM Laboratoire Traitement du Signal et de l'Image (LTSI) - Unité Mixte de Recherche (UMR) 1099, Rennes, France; ^2^ The Australian eHealth Research Centre, Commonwealth Scientific and Industrial Research Organisation (CSIRO), Health and Biosecurity, Brisbane, QLD, Australia; ^3^ School of Mathematical and Physical Sciences, University of Newcastle, Newcastle, NSW, Australia; ^4^ Radiation Oncology, Calvary Mater Newcastle Hospital, Newcastle, NSW, Australia; ^5^ Centre Georges François Leclerc, Dijon, France

**Keywords:** MR-only radiotherapy, dose planning, MRI, deep learning, CT synthesis

## Abstract

**Introduction:**

For radiotherapy based solely on magnetic resonance imaging (MRI), generating synthetic computed tomography scans (sCT) from MRI is essential for dose calculation. The use of deep learning (DL) methods to generate sCT from MRI has shown encouraging results if the MRI images used for training the deep learning network and the MRI images for sCT generation come from the same MRI device. The objective of this study was to create and evaluate a generic DL model capable of generating sCTs from various MRI devices for prostate radiotherapy

**Materials and methods:**

In total, 90 patients from three centers (30 CT-MR prostate pairs/center) underwent treatment using volumetric modulated arc therapy for prostate cancer (PCa) (60 Gy in 20 fractions). T2 MRI images were acquired in addition to computed tomography (CT) images for treatment planning. The DL model was a 2D supervised conditional generative adversarial network (Pix2Pix). Patient images underwent preprocessing steps, including nonrigid registration. Seven different supervised models were trained, incorporating patients from one, two, or three centers. Each model was trained on 24 CT-MR prostate pairs. A generic model was trained using patients from all three centers. To compare sCT and CT, the mean absolute error in Hounsfield units was calculated for the entire pelvis, prostate, bladder, rectum, and bones. For dose analysis, mean dose differences of *D*_99%_ for CTV, *V*_95%_ for PTV, D_max_ for rectum and bladder, and 3D gamma analysis (local, 1%/1 mm) were calculated from CT and sCT. Furthermore, Wilcoxon tests were performed to compare the image and dose results obtained with the generic model to those with the other trained models.

**Results:**

Considering the image results for the entire pelvis, when the data used for the test comes from the same center as the data used for training, the results were not significantly different from the generic model. Absolute dose differences were less than 1 Gy for the CTV *D*_99%_ for every trained model and center. The gamma analysis results showed nonsignificant differences between the generic and monocentric models.

**Conclusion:**

The accuracy of sCT, in terms of image and dose, is equivalent to whether MRI images are generated using the generic model or the monocentric model. The generic model, using only eight MRI-CT pairs per center, offers robust sCT generation, facilitating PCa MRI-only radiotherapy for routine clinical use.

## 1 Introduction

Computed tomography (CT) is generally used as the reference imaging modality for radiation therapy (RT) dose planning (1). However, with the recent emergence of the MRI Linac (a linear accelerator combined with an MRI device) (2), there is a rapidly growing interest in complementing or even replacing CT with MRI in the RT field owing to its superior soft-tissue contrast. MRI is the standard of care for prostate delineation because of its lower volume, which translates into lower doses delivered to adjacent organs at risk (3). Additionally, an MRI-only RT workflow avoids extra imaging radiation to the patient and reduces errors related to intermodality registration (4).

The main challenge with MRI-only RT is that MRI intensity values do not directly correlate with electron densities, which are necessary for accurate dose calculation (4). Several approaches for dose calculation from MRI have been proposed (5). These can be classified into three categories: bulk density, atlas-based, and machine learning methods (including deep learning methods [DLMs]) (6–8). Literature comparisons of these methods reveal that the best results are obtained from generating a synthetic CT (sCT) from MRI using DLMs (7–10). These DLMs can be supervised (using paired data from the same patient) or unsupervised (which does not require paired or registered CT/MRI from the same patient) (8). DLMs can be based solely on generators (ResNet, UNet, etc.) or use the generative adversarial network (GAN) architecture (like conditional GAN, Pix2Pix, and CycleGAN). The unsupervised methods predominantly implement the cycle GAN architecture.

Regardless of the DLM’s architecture, a training step is essential before generating an sCT. The challenge is to produce an accurate sCT from a single training session, irrespective of the MR acquisition device and/or sequence parameters. In routine practice, it would be impractical for each clinical center to conduct a unique training (for each MR sequence and anatomical localization). Most studies on sCT generation (6–8) are monocentric, meaning both training and testing images originate from the same MR device. However, these models are not adaptable to the variability between MR devices in multicenter workflows and thus cannot be universally applied but are instead restricted to specific centers. This is a major limitation of the clinical implementation of sCT.

Few multicenter studies exist in the current state of the art, and they mainly focus on H&N, brain, and pelvis regions. Only four studies (11–14) have used a DLM for pelvis sCT generation in a multicentric context. Three of these were sourced from a gold atlas (15). Two studies used cervix (11) or anorectal data (12) with prostate data. Three adopted the cGAN methods (11–13) [one using the Pix2Pix architecture (11)], and one employed the CycleGAN method with unsupervised training (13).

In the context of generating sCT from MRI specifically for prostate data, there is a notable gap in information regarding the potential to create a generic model. Such a model would enhance the generalizability of MR-to-CT synthesis using pelvis data, thus enabling multicentric data use in both the training and testing phases.

This study aimed to evaluate a generic deep learning (DL) model for MR-to-CT synthesis in prostate MR-only radiotherapy.

## 2 Materials and methods

**Figure 1:**
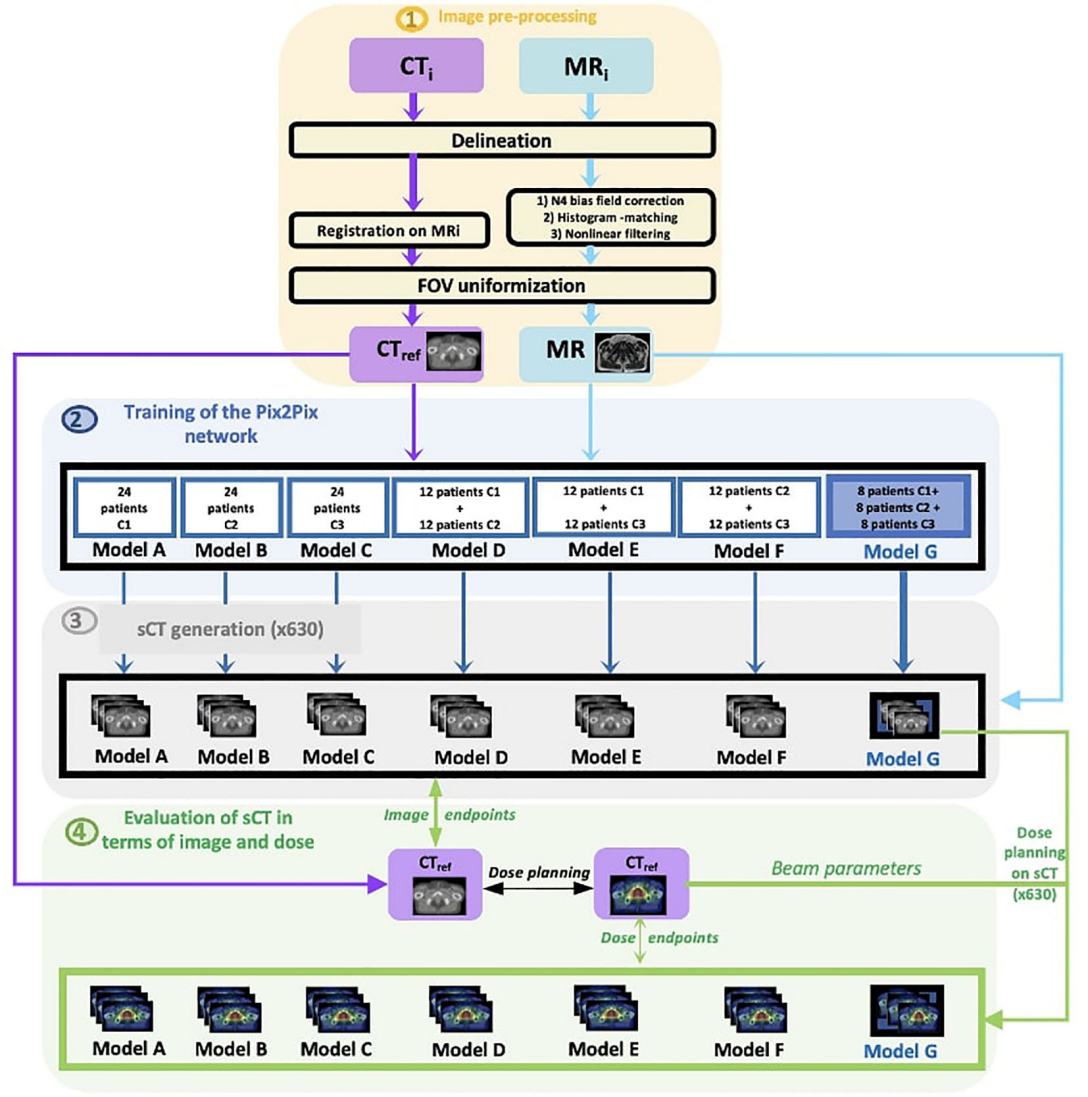
Workflow of the study.

**Figure 1** describes the workflow of the study.

To improve the robustness of the model, the first step involves preprocessing the CT and MR images for each center to standardize the database. For each center, the initial CTs (CTi) and MRs (MRi) were delineated by a radiation oncologist. Subsequently, the CTs were non-rigidly registered to their corresponding MRs (the method used is supervised, then the deformable registration step of the CT/MR pairs is mandatory). The MRs underwent normalization through (1) N4 bias field correction, (2) histogram matching, and (3) nonlinear filtering. Furthermore, each CT and MR image was cropped to retain 8 cm on each side of the prostate, ensuring a consistent FOV for each patient.

The second step focuses on training the Pix2Pix network. Seven distinct models, based on the learning center mixing, were created (with 24 patients per model). The first three models (A, B, C) are composed of patients from a single center; the next three models (D, E, F), which are called the “mixed models,” are composed of patients from two centers; and the last model (model G) is the model comprising patient data from all three centers and is termed the “generic model.”

For the mixed models and generic model, 12 and eight patients were respectively chosen from the pool of 24 cases used in the monocentric model. Likewise, for the test, the six patients used to evaluate the mixed models were the same as the monocentric models.

The third step entails the generation of sCTs. For each model, 30 sCTs are produced in cross-validation, resulting in 210 sCTs per center (630 in total).

The fourth step evaluates the images and doses by comparing the generated sCTs to the reference CT. A dose plan is computed for each reference CT (90 in total). Subsequent dose calculations are conducted using the sCT by using the beam parameters from the CT planning, leading to a total of 630 dose calculations. The dosimetric comparison is achieved by contrasting the dose based on CTref with the dose recalculated on sCT.

For every patient, the image and dose outcomes for the sCTs created with the generic model are compared against the outcomes of other models using a Wilcoxon test.


$$\begin{array}{c} \text{CTi}=\text{initial CT},\text{ MRi}=\text{initial MR},\text{ CTref}=\text{reference CT},\text{ sCT}\\ =\text{synthetic CT},\text{ C}1=\text{center }1,\text{ C}2=\text{center }2,\text{ C}3\\ =\text{center }3,\text{ and FOV}=\text{field of view}. \end{array}$$

### 2.1 Patient data collection

This study included MR and CT images from 90 patients with prostate cancer, which were acquired from three different centers (30 patients per clinical center). **Table 1** presents the data characteristics. The magnetic fields of the MR devices range from 0.35 to 3 T; CT and MR scans were acquired in the RT treatment position. For all patients, the organ delineation on OARs and target volume was performed manually on both CT and MRI, by the radio-oncologists at each center in accordance with the GETUG/RECORAD (16) group recommendation.

**Table 1:** Acquisition parameters of the modalities (CT and MR) for the three centers (C1, C2, and C3).

Number of patients	C1		C2	C3
	30		30	30
CT				
**Manufacturer**	General Electric	Toshiba	General Electric	SIEMENS
**Model**	LightSpeedRT large-bore	Aquilion	LightSpeedRT 16	SOMATOM Confidence
**Slice thickness (mm)**	2.5	2.0	2.5	2
MR				
**Manufacturer**	Siemens		ViewRay Inc.	GE
**Model**	Skyra 3 T		MRIdian	Medical System Optima MR450 w
			0.35 T	1.5 T
**Slice thickness (mm)**	1.6		1.5	1.6
**Sequence type**	T2-weighted		T2/T1-weighted	T2-weighted
**Bandwidth (Hz/pixel)**	250		535	325.508
**TR (ms)**	1200		3.37	1800
**TE (ms)**	102		1.45	87

### 2.2 Image preprocessing

To ensure a smooth workflow and harmonize the data from each center, patient images underwent three preprocessing steps (**Figure 1**, step 1), which are described in the following subsections.

#### 2.2.1 Correction of MRI nonuniformity

To perform the same pre-processing on the entire cohort, regardless of training center or acquisition system manufacturer, the same parameters for correction of MRI nonuniformity were applied to all the data from the three centers.

The T2-weighted scans were preprocessed using the following steps with the Insight Segmentation (17) and Registration Toolkit (ITK) to correct MRI nonuniformity and normalize MRI contrast as described in (18): (1) N4 bias field correction with B-spline fitting (19): parameters [160, 3, 0, 0.5]; convergence: [100 × 100 × 100, 0.001]; shrink factor: (2) MRI contrast normalization using histogram matching with levels: 1024, match points: 7, and a threshold at mean intensity. (3) Nonlinear filtering via anisotropic diffusion (7) with 10 iterations, a time step of 0.03, and a conductance of 1.0. The MR images from the center 1 (C1) had already been pre-processed by Dowling et al. (18). These correction algorithms were performed in addition to vendor algorithms.

#### 2.2.2 Image registration

Furthermore, although the delay between CT and MRI acquisitions was minimized, there were still slight differences in the patient’s anatomy between acquisitions. Since the approach used was supervised, the CT/MRI pairs must be registered in the most optimal way, otherwise, the generation of sCT could be compromised. Therefore, each CT was registered to its corresponding MRI (of the same patient) as described in (9) (metric, normalized cross-correlation; geometric transform, rigid). A structure-guided deformable registration was then computed to ensure bone rigidity while facilitating high-quality bladder and rectum deformable registration (metric, normalized mutual information with 64 bins; geometric transform, B-spline freeform deformation). This registered CT was treated as the ground truth and was termed the reference CT (CTref).

#### 2.2.3 FOV uniformity correction

Each MR and CT image was cropped to maintain a common FOV that extends 8 cm above and below the geometric center of the prostate for the images from each center. Moreover, the FOV was larger for CT than for MRI. This step standardized the FOV size across all centers. The choice of 8 cm was based on clinical criteria, ensuring sufficient tissue for accurate dose calculation. Additionally, B-spline resampling was employed to ensure standardized dimensions of 256 × 256 × 128 for both MR and CT images across all centers.

### 2.3 Network

#### 2.3.1 Pix2Pix network

The 2D supervised model used for this work was Pix2Pix (20), a conditional GAN (cGAN) architecture previously described in a study (20). This architecture comprises two neural networks: a generator and a discriminator. The generator produces sCT images from MR images, whereas the discriminator assesses the quality of the sCT images in comparison to the reference CT image. The training was performed with the Adam optimization algorithm as described by Isola et al. (20). Parameters of the Pix2Pix network, determined from a prior study (18), were set as follows: a learning rate of 2.10^−4^, momentum parameters of 0.5 and 0.999 for β1 and β2, respectively, and 100 epochs. For training our model, a perceptual loss was introduced. The generator network (2D ResNet 9 blocks) aimed to generate sCTs from patient MRIs. The discriminator was a PatchGAN, which takes an sCT image as input and outputs a probability value. This value is almost 1 if the sCT image resembles a genuine CT image and approaches 0 if it looks like a fake CT image. Training proceeded iteratively and halted when the discriminator could no longer accurately discern if the sCTs generated by the creator resembled real or fake CTs (10). In our research, PatchGAN segmented the generated images into 70 × 70 voxel patches. The adversarial loss function of the discriminator is the binary cross-entropy (BCE) (9).

#### 2.3.2 Training data

Overall, seven trainings were performed (**Figure 1**, step 2). The A, B, and C models were composed of patients from a single center, the D, E, and F models, which were called the “mixed models”, were composed of patients from two centers and model G was the model comprising patient data from all three centers and was called the “generic model.” From a database of 30 patients from each center, 24 patients were used for training and six patients in five cross-validation testing. To maintain a consistent number of patients in training, 24 patients from the same center were used for monocenter training (models A, B, and C); 12 patients per center were used for training that included patients from two centers (mixed models D, E, and F); and eight patients per center were used for training that included patients from all three centers (generic model G). For the mixed models and generic model, 12 and eight patients were respectively chosen from the pool of 24 cases used in the monocentric model. Likewise, for the test, the six patients used to evaluate the mixed models were the same as the monocentric models. Indeed, the number constituting the training set has a great impact on the sCT quality (21). However, in this study, only the multicenter impact wants to be studied, therefore the same training size will be used for all seven trainings.

#### 2.3.3 sCT generation

The tests were systematically conducted on all the patients in each center in cross-validation. Overall, 210 sCTs were generated by Pix2Pix for each center (**Figure 1**, step 3).

### 2.4. sCT evaluation

#### 2.4.1 Voxel-wise comparison

The delineations were rigidly propagated from the CTref to the sCT images generated by the 7 training models with each test center image. CTref and generated sCT had the same voxel size to ensure the correct rigid propagation of the contours. To ensure uniform voxel size alignment between the CT and the sCT, we extracted the reference value from each patient’s reference CT and then applied this value to the sCT. The voxel size ranged between 0.9 mm and 1.3 mm for both the sagittal and coronal directions, while it ranged between 1.4 mm and 1.8 mm along the axial direction.

The mean absolute error was computed by comparing corresponding CT and sCT pairs at a voxel level. The global quality of sCT (in terms of image and dose) was evaluated with respect to the patient’s structures (prostate, bladder, rectum, and bones) and entire pelvis by computing (MAE) defined as follows:


(1) $$\text{MAE}=\frac{1}{n}\sum_{i=1}^{n}\left|X_{\text{CTref}}(i)-X_{\text{sCT}}(i)\right|$$

with *n* being the number of voxels, and *X*_CT_(i) and X_sCT_(i) are the intensities of the *i*th voxel in, respectively, the reference and the generated image, in Hounsfield units (HU) for image evaluation or in Gray or percent for dose evaluation.

The ideal MAE value is 0. MAE values were calculated for the following volumes: entire pelvis, bones, prostate, bladder, and rectum.

#### 2.4.2 Dose comparison

Volumetric modulated arc therapy was planned using the RayStation TPS v. 11B using the Collapsed Cone algorithm on a 2 × 2 × 2-mm³ grid. Treatment was delivered at a total dose of 60 Gy to the prostate in 20 fractions of 3 Gy each. The GETUG dose–volume constraints were applied to the organs at risk. The dose plans were redone for each patient of the three centers to have the same methodology for all the cohorts. The optimization and calculation were performed directly on CTref, and the beam parameters used to compute the dose from the CTref images were also used to calculate the dose from the sCT images.

A spatial dose evaluation was conducted via 3D gamma analyses with the Verisoft software, using criteria: local, a dose difference of 1%, a distance to agreement of 1 mm, and a dose threshold of 10% of the prescription dose. This analysis compared the dose distributions from CTref and sCTs.

Dose distributions were analyzed through absolute dose differences (1) at *D*_99%_ for CTV, *V*_95%_ for PTV, and *D*_max_ for rectum and bladder. This voxel-wise dose difference is termed MAE in dose terms.

#### 2.4.3 Statistical analyses

Wilcoxon signed-rank tests were conducted to compare the image and dose results. For MAE (image and dose), this test compared the MAE of the generic model with that of the other models and among each test center.

## 3 Results

### 3.1 Voxel-wise comparison

**Figure 2:**
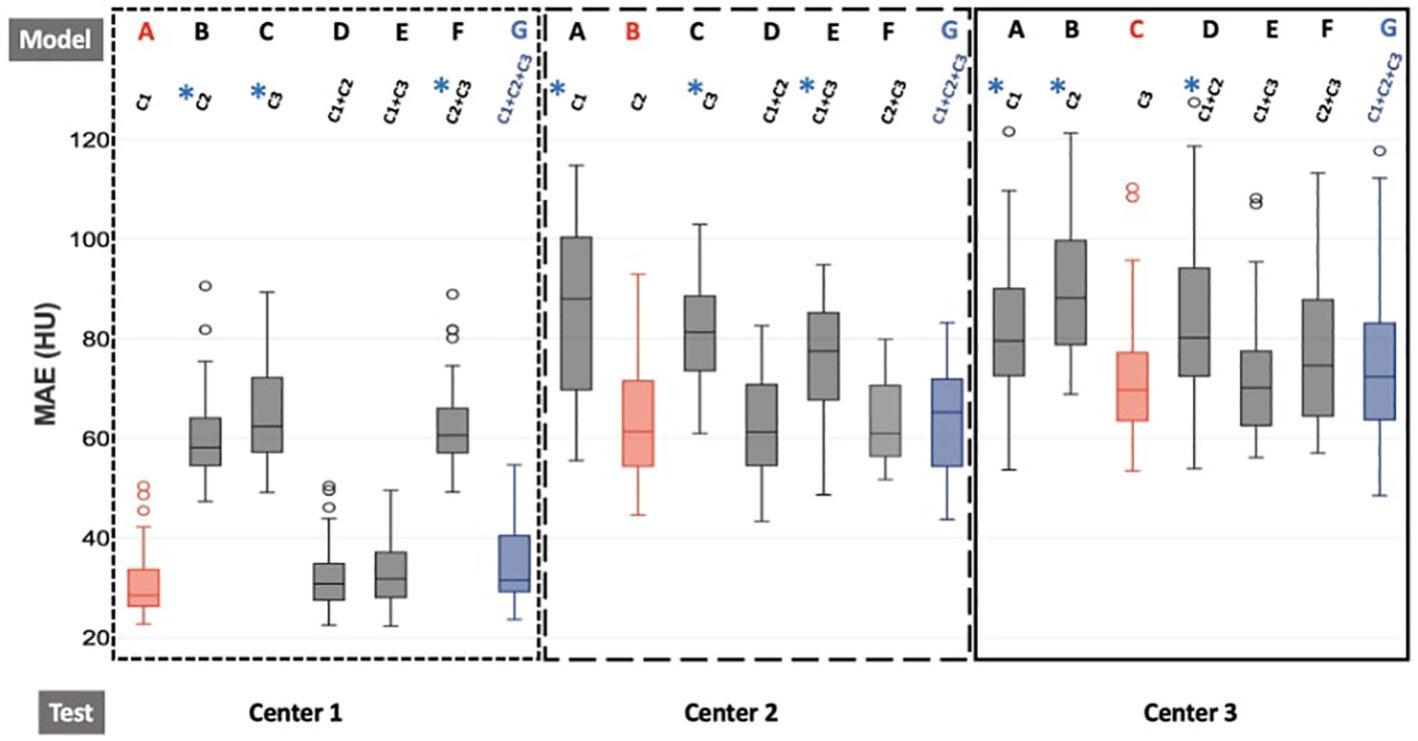
Boxplot of MAE results for the different training models for each test center for the entire pelvis. The dotted line represents the results for center 1, the larger dotted line for center 2, and the solid line for center 3. Furthermore, red boxes indicate the monocentric models (model A/test C1, model B/test C2, model C/test C3), and blue boxes represent the generic model (model G/test C1 or C2 or C3). For each center, the seven models A, B, C, D, E, F, and G are trained with C1, C2, C3, C1+C2, C1+C3, C2+C3, and C1+C2+C3, respectively. The generic model (G) is our reference model. Wilcoxon tests were used to compare the generic model to the other models. ^*^ *p*-value< 0.05, significant differences.

**Figure 2** presents the MAE results for the entire pelvis across the seven models (A–G), based on the training. Additional figures present the MAE results for the prostate, bladder, rectum, and bones.

The average MAE values in the entire pelvis were lower than 90 HU for all seven training models of each center and lower than 80 HU for the monocentric (model A/test C1, model B/test C2, model C/test C3) and generic models of each center (model G test C1 or C2 or C3). For the generic model, 36 HU, 70 HU, and 75 HU were obtained for centers 1, 2, and 3, respectively. For each center, when the data used for the test come from the same center as the data used for training, the results were never significantly different from the generic model.

The average MAE values in the prostate were lower than 40 HU for all seven models of each center and lower than 25 HU for the monocentric (model A/test C1, model B/test C2, model C/test C3) and generic models of each center (model G/test C1 or C2 or C3). For the generic model, 17 HU, 16 HU, and 21 HU were obtained for centers 1, 2, and 3, respectively. For each center, when the data used for the test come from the same center as the data used for training, the results are never significantly different from the generic model. However, often, when the test data come from a center whose data were not used for training, the results are significantly different from the results of the generic model (higher MAE). For example, for center 1, the models B (trained with C2), C (trained with C3), and F (trained with C2+C3) have significantly higher MAE than the generic model (17 HU), with mean MAE respectively equal to 33 HU, 27 HU, and 22 HU, but the results for the models A (trained with C1), D (trained with C1+C2), and E (trained with C1+C3) were not significantly different, with mean MAE respectively equal to 17 HU, 20 HU, and 17 HU.

The average MAE values in the bladder for all seven training models of each center were lower than 60 HU and lower than 25 HU for the monocentric (model A/test C1, model B/test C2, model C/test C3) and generic models of each center (model G/test C1 or C2 or C3). For the generic model, 16 HU, 27 HU, and 18 HU were obtained for centers 1, 2, and 3, respectively.

For the generic model, 17 HU, 16 HU, and 21 HU were obtained for centers 1, 2, and 3, respectively. For each center, when the data used for the test comes from the same center as the data used for training, the results were never significantly different from the generic model. However, often, when the test data comes from a center whose data was not used for training, the results are significantly different from the results of the generic model (higher MAE). For example, for center 1, model B (trained with C2), C (trained with C3), and F (trained with C2+C3) had significantly higher MAE than the generic model (16 HU), with respectively mean MAE equal to 60, 22, and 40 HU, but the results for the models A (trained with C1), D (trained with C1+C2), and E (trained with C1+C3) were not significantly different, with mean MAE respectively equal to 16, 17, and 17 HU.

The average MAE values in the rectum were between 40 HU and 100 HU for all models, and lower than 60 HU for the monocentric and generic models, which was superior to the other soft tissues. For the generic model, 45 HU, 45 HU, and 52 HU were obtained for centers 1, 2, and 3, respectively.

For center 1, unlike the results for the prostate and bladder, there were no models with significantly different MAE values from the values obtained for the generic model. However, for center 2, model C (trained with C3) had significantly higher MAE than the generic model, with, respectively, mean MAE equal to 58 and 45 HU. For center 3, models B (trained with C2) and D (trained with C1+C2) had significantly higher MAE than the generic model, with, respectively, mean MAE equal to 77, 68, and 55 HU.

The average MAE values in the bones were between 100 HU and 275 HU for all models, which was superior to the other structures. For the generic model, 140 HU, 220 HU, and 240 HU were obtained for centers 1, 2, and 3, respectively.

For center 1, there was a great disparity in results between the models. Indeed, for the monocentric models A (trained with C1), D (trained with C1+C2), and E (trained with C1+C3), i.e., the models when the data used for the test come from the same center as the data used for training, the average value of the MAE was close to the MAE of the generic model with 110 HU, 120 HU, 122 HU, or 138 HU, respectively. On the other hand, for models B (trained with C2), C (trained with C3), and F (trained with C2+C3), i.e., when the test data come from a center whose data were not used for training, the average values of the MAE were significantly higher, with respectively 180, 255, and 178 HU.

For the three centers, the MAE results for monocentric models (model A/test C1, model B/test C2, model C/test C3) were not significantly different from the generic model for each structure.

**Figure 3:**
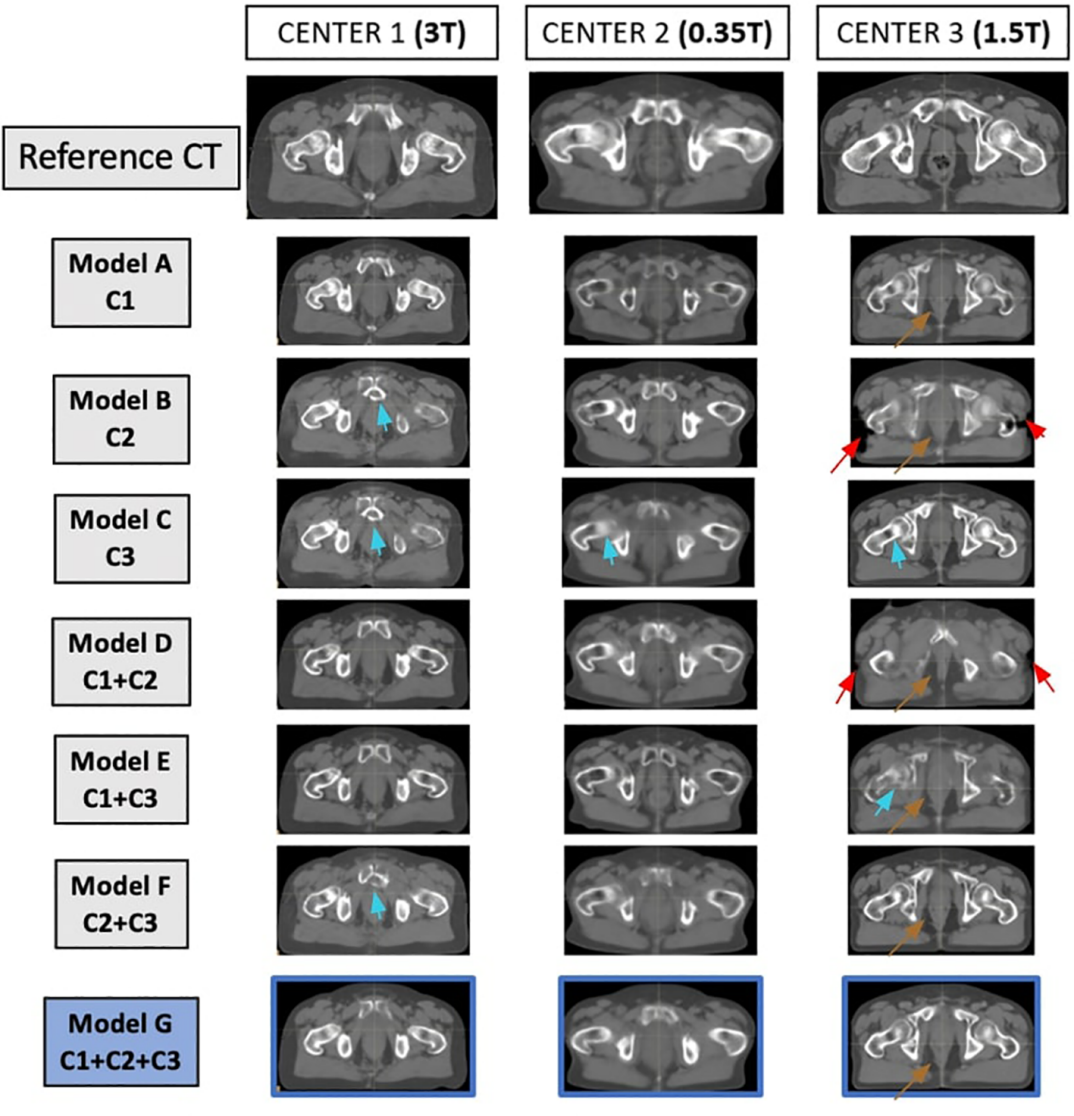
Matched reference CT and synthetic CT (sCT) for each model, for one patient at each center. On the first line, from left to right, the reference CTs of one patient from center 1, one patient from center 2, and one patient from center 3 are represented. For each patient in each center, the sCT generated with each model is represented. The sCT images of each model represent the same slice as the reference CT. Arrows show limitations of the sCT generation (red arrow for misgeneration of the external contour, brown arrow for misgeneration of air pockets, blue arrows for misgeneration of bones).

For a visual comparison, **Figure 3** demonstrates the reference CT and sCT for the seven models for one patient from each center. The sCT of the patient from center 3 exhibits more artifacts, unlike the sCTs of the patients from centers 1 and 2.

### 3.2 Dose comparison

**Figure 4:**
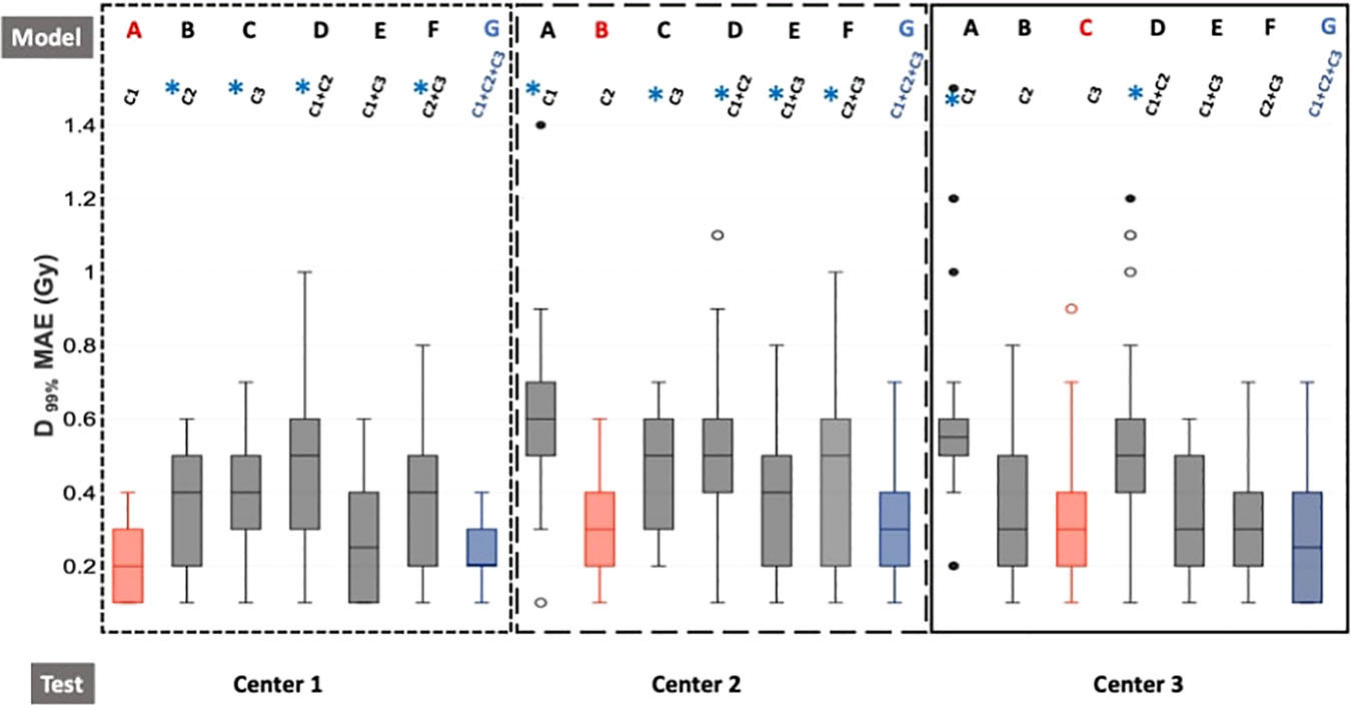
Boxplot of dose absolute errors for *D*_99%_ of the CTV. The dotted line represents the results for center 1, the larger dotted line for center 2, and the solid line for center 3. Furthermore, red boxes indicate the monocentric models (model A/test C1, model B/test C2, model C/test C3), and blue boxes represent the generic model (model G/test C1 or C2 or C3). For each center, the seven models A, B, C, D, E, F, and G are trained with C1, C2, C3, C1+C2, C1+C3, C2+C3, and C1+C2+C3, respectively. The generic model (G) is our reference model. Wilcoxon tests were used to compare the generic model to the other models. ^*^ *p*-value< 0.05, significant differences.

**Figure 4** presents the absolute dose differences for the *D*_99%_ of the CTV, and **Supplementary Table S1** the absolute dose differences for all the DVH indicators considered.

For center 1, models B (*trained with* C2), C (*trained with* C3), D (*trained with* C1+C2), and F (*trained with* C2+C3) showed significantly higher absolute dose differences than the generic model G, while models A (*trained with* C1) and E (*trained with* C1 + C3) showed nonsignificant differences. For center 2, results from model G had significantly lower MAEs than the other models except for model B (C2). For center 3, the generic model G had an MAE significantly lower than models A (*trained with* C1) and D (*trained with* C1 + C2) and nonsignificant differences with other models. The average MAE values for the *D*_99%_ of the CTV were lower than 0.6 Gy for all seven models of each center and lower or equal to 0.3 Gy for the monocentric (model A/test C1, model B/test C2, model C/test C3) and generic models of each center (model G/test C1 or C2 or C3).

For the generic model, 0.2 Gy, 0.3 Gy, and 0.3 Gy were obtained for centers 1, 2, and 3, respectively.

Unlike the image evaluation (MAE in HU), when the data used for the test come from the same center as the data used for training, the results were sometimes significantly different from the generic model. However, the results obtained for the generic model of each center (model G/test C1 or C2 or C3) were never significantly different from those obtained for the monocentric model of each center (model A/test C1, model B/test C2, model C/test C3).

**Figure 5:**
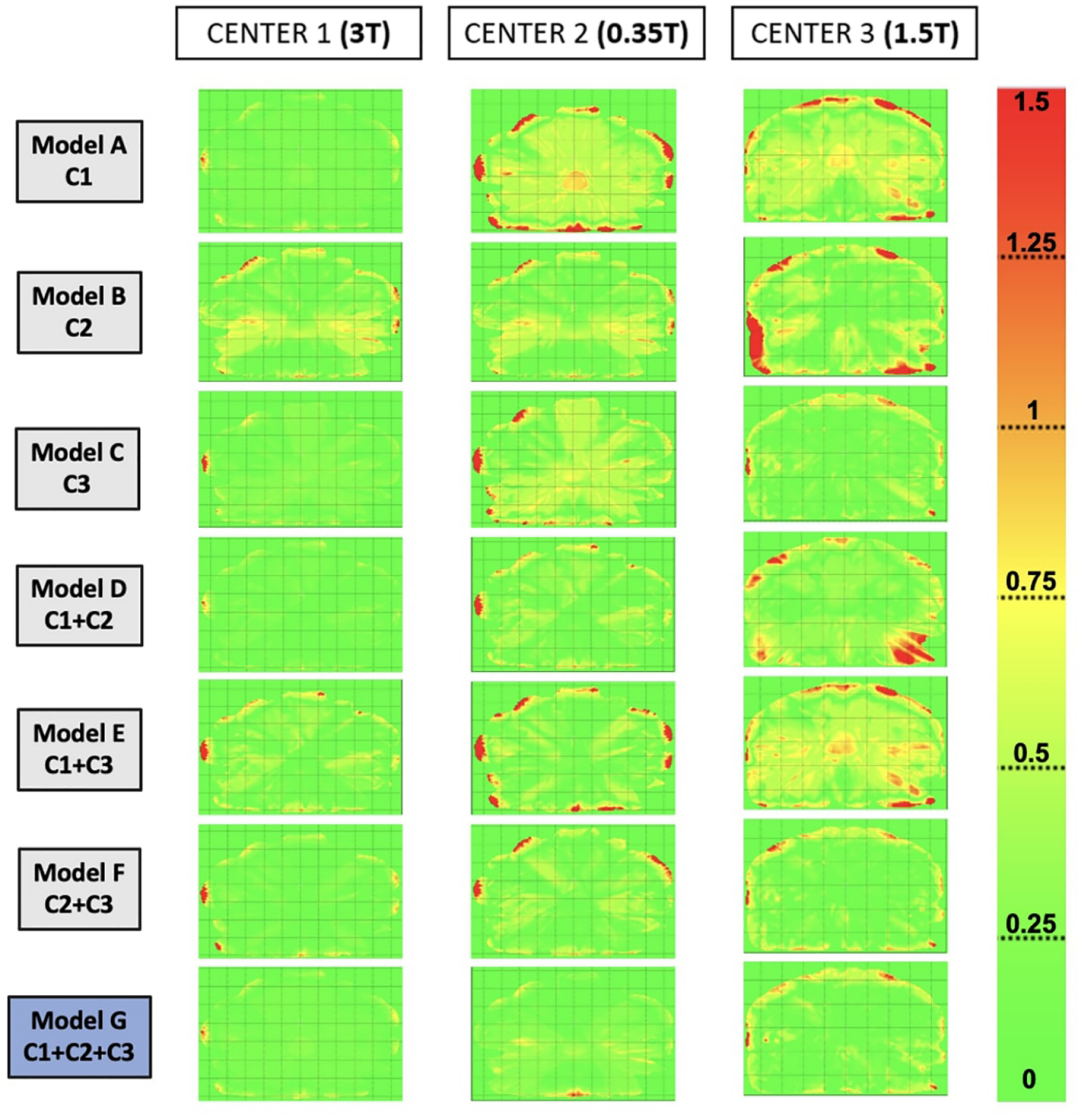
Gamma maps were obtained by comparing the dose distribution on reference CT to the dose distribution on synthetic CT (sCT) for each model, for one patient of each center. The selected patients are the same as **Figure 3**. The gamma criteria are local, 1%/1 mm, and 10% dose threshold.

**Table 2** presents the gamma pass-rate values for different training cohorts at each test center, and **Figure 5** represents gamma maps obtained by comparing reference CT to synthetic CT (sCT) for each model for one patient (the same as **Figure 3**) at each center. For center 1, there were no significant differences between the gamma pass-rate results of the different models. For center 2, the gamma values were significantly lower for models A (*trained with* C1) and B (*trained with* C1 + C3). For each of these models with lower gamma values, there were no patients from center 2. For center 3, the gamma pass-rate values were significantly higher for model C (*trained with* C3), model F (*trained with* C2 + C3), and the generic model (G). Furthermore, for all the centers, the generic model did not show significantly lower results than the other models from the same center.

**Table 2:** Gamma pass-rate values comparing the dose distribution on reference CT with the dose distribution on sCTs.

Number of centers in the train	Models	Gamma pass rate (%)		
		Test C1	Test C2	Test C3
**1**	Model A (C1)	**96.5 ± 1.4**	85.9 ± 10.1^*^	96.1 ± 2.7
	Model B (C2)	96.2 ± 2.4	**97.9 ± 1.4**	96.7 ± 1.7
	Model C (C3)	96.5 ± 2.0	97.3 ± 1.5	**98.7 ± 0.9**
**2**	Model D (C1+C2)	96.4 ± 1.2	98.1 ± 2.2	96.2 ± 3.7^*^
	Model E (C1+C3)	96.5 ± 1.1	94.7 ± 4.5^*^	92.2 ± 5.6^*^
	Model F (C2+C3)	95.8 ± 2.7	98.0 ± 2.0	98.3 ± 1.6
**3**	**Model G (C1+C2+C3)**	**96.3 ± 1.3**	**98.1 ± 1.5**	**98.4 ± 1.4**

The gamma criteria are local, 1%/1 mm, and 10% dose threshold.

The values are expressed as mean ± standard deviation. The Wilcoxon’s test was used to compare the gamma pass rate of the generic model with those of the other models.

^*^p-value< 0.05—significant difference.

Red cases indicate the monocentric models (model A/test C1, model B/test C2, model C/test C3) and blue cases represent the generic model (model G/test C1 or C2 or C3).

## 4 Discussion

This study investigates a DLM’s training with different center mixing for pelvic sCT image generation from various MRI devices. Different models were computed for each of the three test centers (**Figure 1**). The results of each model were evaluated and compared using image and dose endpoints. Previous studies (7–9) have already shown that DLMs allow the creation of accurate sCT images with monocentric data (data from the same center in both the model and test). However, the question persists: can these models accurately generate sCTs from any MRI device? Many have speculated about the network’s inability to perform this generation, hypothesizing that each MRI has distinct characteristics (magnetic field, sequence parameters, etc.). Without training on images from the same MRI device, the generation of sCT might be compromised.

The monocentric model (model A/test C1, model B/test C2, model C/test C3) is considered the model that can obtain the most accurate sCT generation, given that for training and testing, data from the same center were used. Our objective was therefore to get as close as possible to these “ideal” values.

For the generic model composed of patients from each center, the image and dose results were not significantly different from the results obtained with the monocentric models even though there were only eight patients from each center (24 patients in monocentric models). The results with the generic model were also not significantly different from the mixed cohort with models composed of patients from the same center as those of the test center.

Centers 2 and 3 had higher MAE values than center 1, as depicted in **Figure 2**. This discrepancy can be attributed to the data from center 1 being first registered using an atlas method, followed by the same method used for centers 2 and 3. Consequently, the data from center 1 were better registered than the data from centers 2 and 3. The quality of the CT-MR registration plays a crucial role in the successful generation of sCT, with a supervised approach.

In the literature, different MAE results were obtained using similar architectures, ranging from 36 HU to 55 HU (7, 8). Moreover, few studies have employed a Pix2Pix architecture for prostate sCT generation (11, 22). In the Fetty et al. study (11), the MAE for the entire pelvis was 41 HU, and it was 54 HU in the Cusumano et al. study (22). In comparison, the MAE for the entire pelvis for centers 1, 2, and 3 were 31 HU, 85 HU, and 72 HU, respectively. Several factors can explain these findings. The deformable registration method for center 1 differs from the other two centers. For center 2, the images originated from an MRI Linac 0.35 T, TRUFI sequence, and the image quality appeared different, with lower contrast compared to the other two centers. For center 3, most CTs were conducted with contrast agents during the excretory phase, and pretreatment was carried out to address this issue.

Mixed models, consisting of two centers, were conducted (trained with C1 + C2, C1 + C3, and C2 + C3). These models were tested at each center. The results demonstrate that when the model does not include any patients from the test center, the generated sCTs appear visually degraded (**Figure 3**), and the MAE values rise significantly (**Figure 2**). The image quality derived from these models had visual differences compared to generic models and showed significant differences in terms of image quality (**Figure 2**).

For the generic model (model G) comprising patients from each center, the image and dose results were not significantly different from those obtained with the reference monocentric models, even though there were only eight patients from each center (24 patients in monocentric models). The results with the generic model were also not significantly different from the mixed models (trained with two centers, including the considered center, **Figure 2**).

In this study, we demonstrated that the image and dose differences were significantly lower when both the training and the test included patients from the same center.

Regarding studies in the literature that address the generation of sCT for the pelvic region, the results in terms of MAE range between 35 HU and 66 HU. The dose differences on the PTV range between 0.7% and 1.5%. Compared to the literature, our generic model for center 1 presents a lower MAE than the other studies.

This work has highlighted some limitations of the DL network (not just the Pix2Pix architecture) and the evaluation of the sCTs. At times, the image results were satisfactory (when compared to the literature values); however, the sCT image contained artifacts that could jeopardize the accuracy of dose calculation. Thus, a visual inspection is always necessary, and other metrics for evaluating sCT should be considered.

Indeed, we can see in **Figure 3** that artifacts were sometimes present in the images. The calculations of image metrics being averages over a large volume, the differences linked to artifacts do not necessarily modify the average value of MAE obtained, which then seems to confirm the good quality of the sCTs generated. However, in the dosimetric analysis, these artifacts on the images will induce a large dosimetric difference, as shown in **Figure 5**.

MAE results shown in the **Supplementary Figures** indicated that the proposed models, with supervised cGAN-based, could precisely estimate soft tissue HU values but had larger errors in reproducing air-like air pockets in the rectum (brown arrows) and bone (blue arrows). There are a few possible reasons. First, air and bone are both barely visible in MR images due to weak signals, making their HU prediction challenging. Second, registration errors between the MR and CT images would have more impact on the intensity mapping of end-of-range voxels than for soft tissue voxels. Misregistration can cause air and bone tissue boundaries to be shifted, introducing intensity mapping errors. However, misregistration within the soft tissue itself does not have a large impact on the intensity mapping. The red arrows in **Figure 3** represent the misgeneration of the external contour.

The misgeneration of the external contour on the sCT (shown by the red arrows in **Figure 3**) appears visually but also has a dosimetric consequence, as shown in **Figure 5**. Indeed, for the patient in center 3, the gamma maps show a great difference in dose for the areas where the external contour has not been generated. The gamma pass rate results were also affected.

All evaluations were conducted by comparing the reference CT to the sCT. However, to advance in an MRI-only RT workflow, we need clinically integrated tools for quality assurance of sCT without any reference to CT (23).

Furthermore, in our study, we did not include patients with atypical anatomy. For instance, no patient in our cohort had a hip prosthesis (neither in training nor in testing), so we are unsure of the sCT outcome in such cases.

To achieve low image and dose uncertainties for generating sCT, several factors must be considered. First, the field of view is crucial. During this study, several images were excluded due to a limited field of view on the MR image, specifically when the FOV was less than 10 cm. Although generating sCT with a limited field of view is feasible, it is more efficient with a broader one as it provides more image information. Additionally, generating sCTs for patients with overly full or entirely empty bladders proved challenging, as we identified generation artifacts for these patients. Lastly, for patients with excessive gas in the rectum, evaluating the sCT was complicated in the rectal area.

Firstly, when the bladder of the same patient did not have the same filling on CT and on MRI, the deformable registration was more complicated, and there remained differences between CT and MRI, but it was the structures of the CT that were used to evaluate sCT generated from MRI. This is the first limitation. Secondly, for most patients, the bladder filling is not completely empty or filled. Therefore, the training of the network is done mostly with patients having an intermediate bladder. However, when the sCT is generated for a patient with a completely empty or filled bladder, the generation can prove to be more problematic. On the sagittal plane, we noticed artifacts on the bladder for patients with extreme bladder sizes (totally empty or full).

For patients with a difference in gas pockets in the rectum between CTref and sCT, evaluation in terms of an image in the rectum may be problematic. Indeed, if the patient does not present gas pockets in the rectum during the MR acquisition, this was the case during the CT acquisition. Then the sCT generated from the MRI should not show pocket gas. However, as the MAE is a comparison of HU between CT and sCT, the differences in gas pockets in the rectum will be interpreted as misgeneration, whereas they are simply due to the anatomical difference between CT and MRI. In the same way, if it is on the CT that there is pocket gas in the rectum and not on the MRI, the problem will be the same but it will be less so because we have observed that deep learning networks experience difficulty generating gas pockets in the rectum, probably because in the training data, pockets of gas are present randomly depending on the patients. These visual observations are confirmed by the results in terms of images. The MAE values are higher for the rectum than for the prostate or the bladder, for example.

This study reaffirmed our assumptions about the challenges of using supervised single-center training for generating sCT from MRIs from different centers. We now advocate for the creation of a generic model. Nevertheless, there is still much to investigate further. For instance, the labor-intensive image registration step might be bypassed with unsupervised methods. Indeed, in the pelvis, often, the patient’s anatomy has changed between the acquisition of CT and MRI, especially concerning the pocket gas in the rectum and bladder filling. Indeed, MRI and CT cannot be acquired at the same time to maintain the same anatomy. Supervised generation requires a perfect registration of CT/MRI pairs to obtain accurate sCT. The results between the different centers were impacted when the deformable registration was not perfect. To overcome this problem, an unsupervised method can be considered, which permits to overcome deformable registration.

Moreover, the interslice artifacts mentioned in the results are mainly due to the use of a 2D method, like most of today’s synthesis methods. A potential solution is the use of a 3D method, even if, 3D learning requires more patients in the training cohort than the 2D method and has a higher computational cost.

Other perspectives for improving the quality of sCT can subsequently be considered, such as the development of methods to reduce artifacts to allow better training and improve generation, or even training on more data from other centers in an international partnership.

Finally, the generic model should be tested on an MRI from another center that has never been in a training cohort.

For optimal use of the generic model across any center, it is recommended to maintain a standardized MRI acquisition procedure.

## 5 Conclusion

To produce accurate sCT images from various MRI devices with the aim of prostate dose planning in RT, the generic DLM offers comparable image and dose calculation uncertainties to monocentric studies. The next step is implementing a generic model in the clinical practice of MR-only radiotherapy, thus eliminating the need for reference CT acquisition.

## Data availability statement

The original contributions presented in the study are included in the article/**Supplementary Material**. Further inquiries can be directed to the corresponding author.

## Author contributions

ST: Writing – original draft, Writing – review & editing, Methodology, Investigation, Formal analysis. BT: Writing – original draft, Formal Analysis. JN: Writing – original draft, Writing – review & editing, Supervision. CH: Writing – original draft, Formal Analysis. PL: Writing – original draft, Formal analysis. EC: Writing – original draft, Formal analysis. HC: Writing – original draft. JL: Writing – original draft. PG: Writing – original draft. JD: Writing – original draft. OA: Writing – original draft. IB: Writing – original draft, Resources. LM: Writing – original draft, Resources. AB: Writing – original draft, Resources. RD: Writing – original draft, Supervision. CL: Writing – original draft, Supervision. AB: Writing – original draft, Writing – review & editing, Supervision.

## Supplementary material

Supplementary Figure 1 Boxplot of MAE results for the different models for each test center for the prostate. The dotted line represents the results for center 1, the larger dotted line for center 2, and the solid line for center 3. Furthermore, red boxes indicate the monocentric (Model A/Test C1, Model B/Test C2, Model C/Test C3), and blue boxes represent the generic model (Model G/Test C1 or C2 or C3). For each center, the 7 models A, B, C, D, E, F, and G are trained with C1, C2, C3, C1+C2, C1+C3, C2+C3, C1+C2+C3 respectively. The generic model (Model G) is our reference model, Wilcoxon tests were used to compare the generic model to the other models. *Significant differences were considered at a p-value< 0.05.

Supplementary Figure 2 Boxplot of MAE results for the different models for each test center for the bladder. The dotted line represents the results for center 1, the larger dotted line for center 2, and the solid line for center 3. Furthermore, red boxes indicate the monocentric (Model A/Test C1, Model B/Test C2, Model C/Test C3), and blue boxes represent the generic model (Model G/Test C1 or C2 or C3). For each center, the 7 models A, B, C, D, E, F, and G are trained with C1, C2, C3, C1+C2, C1+C3, C2+C3, C1+C2+C3 respectively. The generic model (Model G) is our reference model, Wilcoxon tests were used to compare the generic model to the other models. *Significant differences were considered at a p-value< 0.05.

Supplementary Figure 3 Boxplot of MAE results for the different models for each test center for the rectum. The dotted line represents the results for center 1, the larger dotted line for center 2, and the solid line for center 3. Furthermore, red boxes indicate the monocentric (Model A/Test C1, Model B/Test C2, Model C/Test C3), and blue boxes represent the generic model (Model G/Test C1 or C2 or C3). For each center, the 7 models A, B, C, D, E, F, and G are trained with C1, C2, C3, C1+C2, C1+C3, C2+C3, C1+C2+C3 respectively. The generic model (Model G) is our reference model, Wilcoxon tests were used to compare the generic model to the other models. *Significant differences were considered at a p-value< 0.05.

Supplementary Figure 4 Boxplot of MAE results for the different models for each test center for bones. The dotted line represents the results for center 1, the larger dotted line for center 2, and the solid line for center 3. Furthermore, red boxes indicate the monocentric (Model A/Test C1, Model B/Test C2, Model C/Test C3), and blue boxes represent the generic model (Model G/Test C1 or C2 or C3). For each center, the 7 models A, B, C, D, E, F, and G are trained with C1, C2, C3, C1+C2, C1+C3, C2+C3, C1+C2+C3 respectively. The generic model (Model G) is our reference model, Wilcoxon tests were used to compare the generic model to the other models. *Significant differences were considered at a p-value< 0.05.

Supplementary Table 1 Mean values of volume and dose difference calculated between CT and sCT for all the DVH indicators considered. Absolute dose values were reported in Gy for all the parameters investigated except for *V*_95%_ of PTV where the volume percentage difference was considered. The values are expressed as mean ± standard deviation. Red cases indicate the monocentric models (model A/test C1, model B/test C2, model C/test C3), and blue cases represent the generic model (model G/test C1 or C2 or C3).
