# Supplementary material for: A deep learning model to generate synthetic CT for prostate MR-only radiotherapy dose planning: a multicenter study
Source: Front Oncol. 2023 Nov 28;13:1279750. doi: 10.3389/fonc.2023.1279750 (PMC10713720; doi:10.3389/fonc.2023.1279750)
Supplement: Supplementary Figure 1 — Boxplot of MAE results for the different models for each test center for the prostate. The dotted line represents the results for center 1, the larger dotted line for center 2, and the solid line for center 3. Furthermore, red boxes indicate the monocentric (Model A/Test C1, Model B/Test C2, Model C/Test C3), and blue boxes represent the generic model (Model G/Test C1 or C2 or C3). For each center, the 7 models A, B, C, D, E, F, and G are trained with C1, C2, C3, C1+C2, C1+C3, C2+C3, C1+C2+C3 respectively. The generic model (Model G) is our reference model, Wilcoxon tests were used to compare the generic model to the other models. *Significant differences were considered at a p-value< 0.05. [file DataSheet_1.docx]

# Additional Figures


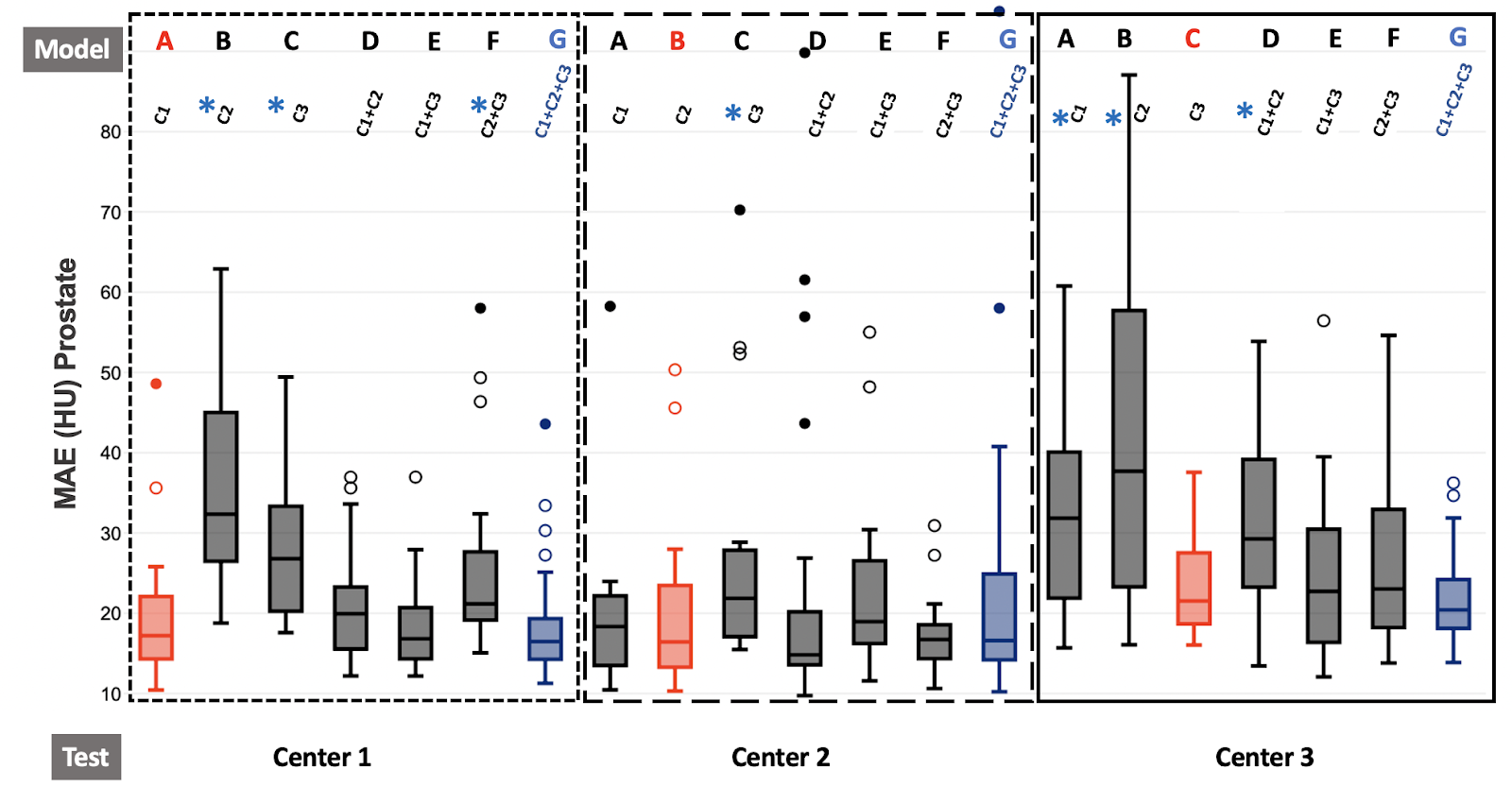


*Additional figure 1. Boxplot of MAE results for the different models for each test center for the prostate*

*The dotted line represents the results for center 1, the larger dotted line for center 2, and the solid line for center 3. Furthermore, red boxes indicate the monocentric (Model A/Test C1, Model B/Test C2, Model C/Test C3) and blue boxes represent the generic model (Model G/Test C1 or C2 or C3).*

*For each center, the 7 models A, B, C, D, E, F, G are trained with C1, C2, C3, C1+C2, C1+C3, C2+C3, C1+C2+C3 respectively.*

*The generic model (Model G) is our reference model, Wilcoxon tests was used to compare the generic model to the other models.*

**Significant differences were considered at a p-value < 0.05.*


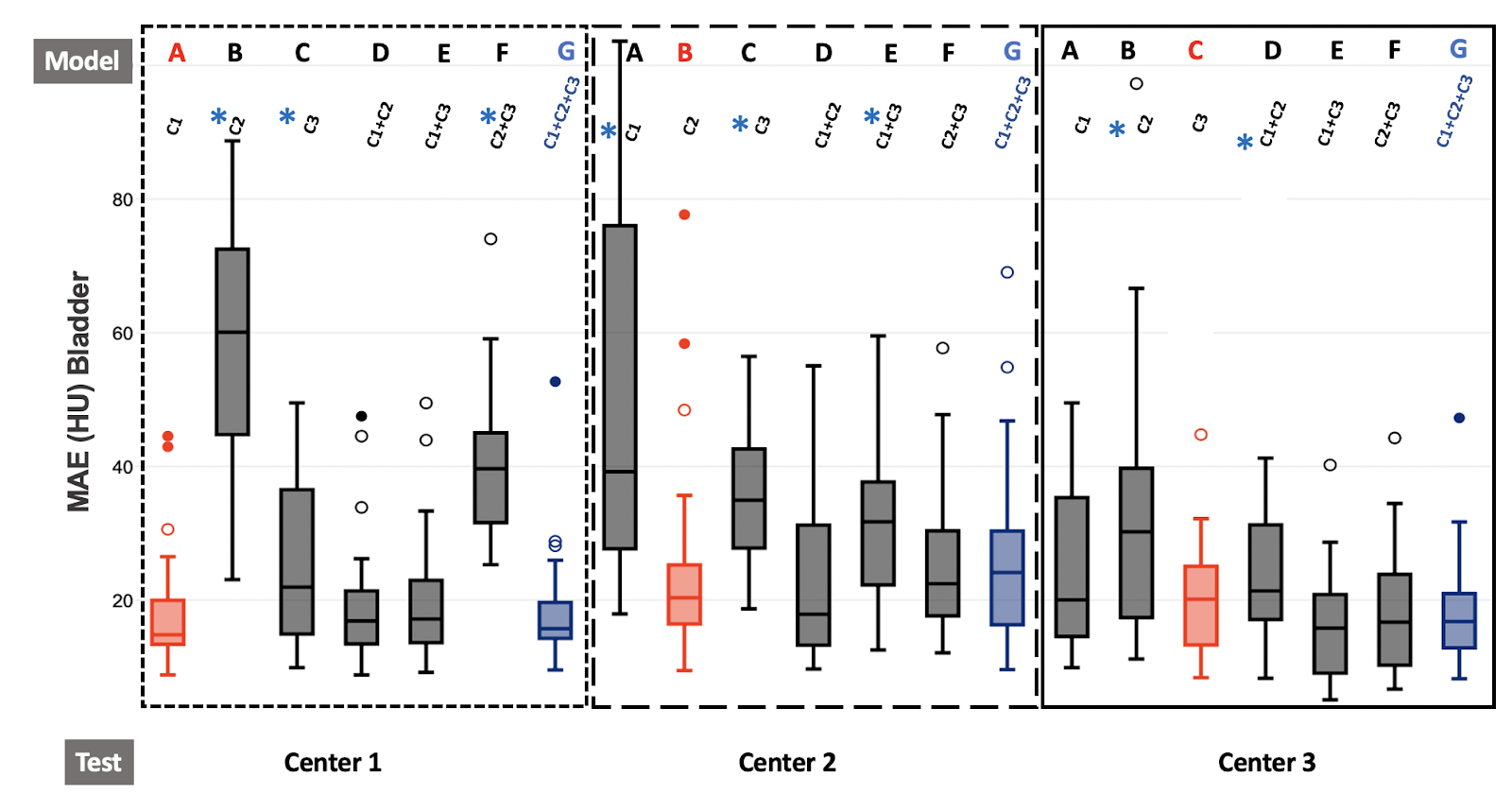


*Additional figure 2. Boxplot of MAE results for the different models for each test center for the bladder*

*The dotted line represents the results for center 1, the larger dotted line for center 2, and the solid line for center 3. Furthermore, red boxes indicate the monocentric (Model A/Test C1, Model B/Test C2, Model C/Test C3) and blue boxes represent the generic model (Model G/Test C1 or C2 or C3).*

*For each center, the 7 models A, B, C, D, E, F, G are trained with C1, C2, C3, C1+C2, C1+C3, C2+C3, C1+C2+C3 respectively.*

*The generic model (Model G) is our reference model, Wilcoxon tests was used to compare the generic model to the other models.*

**Significant differences were considered at a p-value < 0.05.*


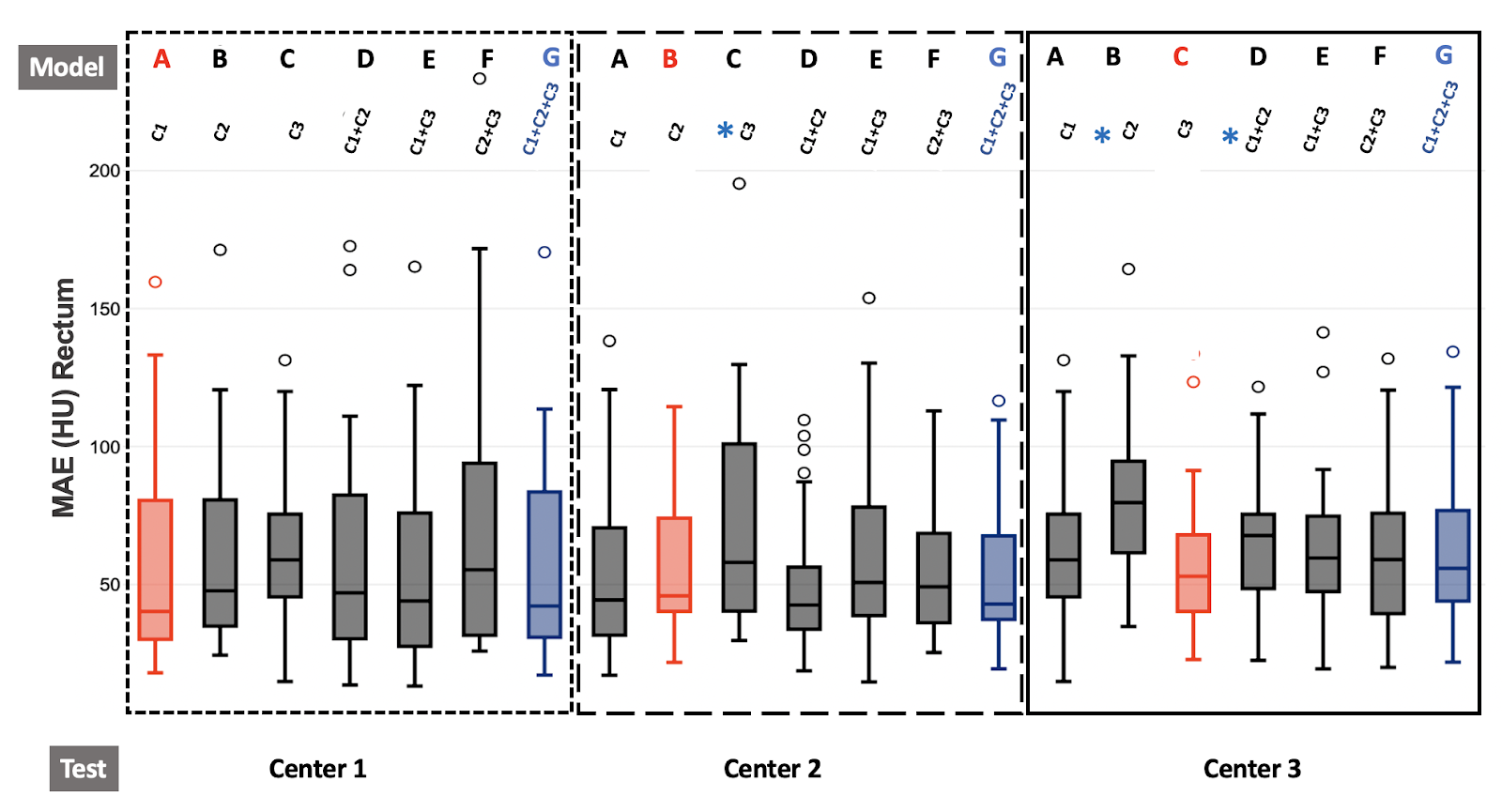


*Additional figure 3. Boxplot of MAE results for the different models for each test center for the rectum*

*The dotted line represents the results for center 1, the larger dotted line for center 2, and the solid line for center 3. Furthermore, red boxes indicate the monocentric (Model A/Test C1, Model B/Test C2, Model C/Test C3) and blue boxes represent the generic model (Model G/Test C1 or C2 or C3).*

*For each center, the 7 models A, B, C, D, E, F, G are trained with C1, C2, C3, C1+C2, C1+C3, C2+C3, C1+C2+C3 respectively.*

*The generic model (Model G) is our reference model, Wilcoxon tests was used to compare the generic model to the other models.*

**Significant differences were considered at a p-value < 0.05.*


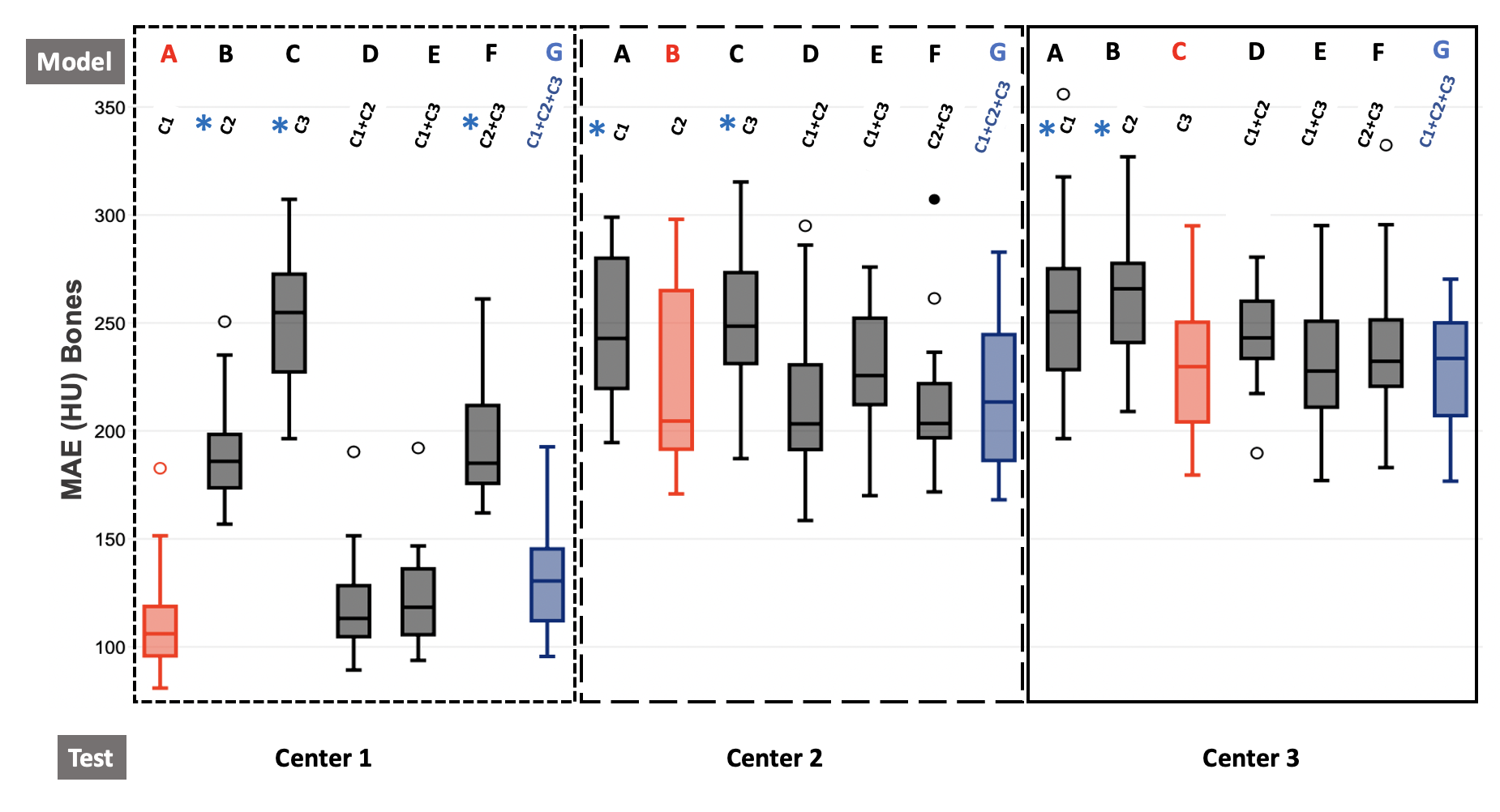


*Additional figure 4. Boxplot of MAE results for the different models for each test center for bones*

*The dotted line represents the results for center 1, the larger dotted line for center 2, and the solid line for center 3. Furthermore, red boxes indicate the monocentric (Model A/Test C1, Model B/Test C2, Model C/Test C3) and blue boxes represent the generic model (Model G/Test C1 or C2 or C3).*

*For each center, the 7 models A, B, C, D, E, F, G are trained with C1, C2, C3, C1+C2, C1+C3, C2+C3, C1+C2+C3 respectively.*

*The generic model (Model G) is our reference model, Wilcoxon tests was used to compare the generic model to the other models.*

**Significant differences were considered at a p-value < 0.05.*


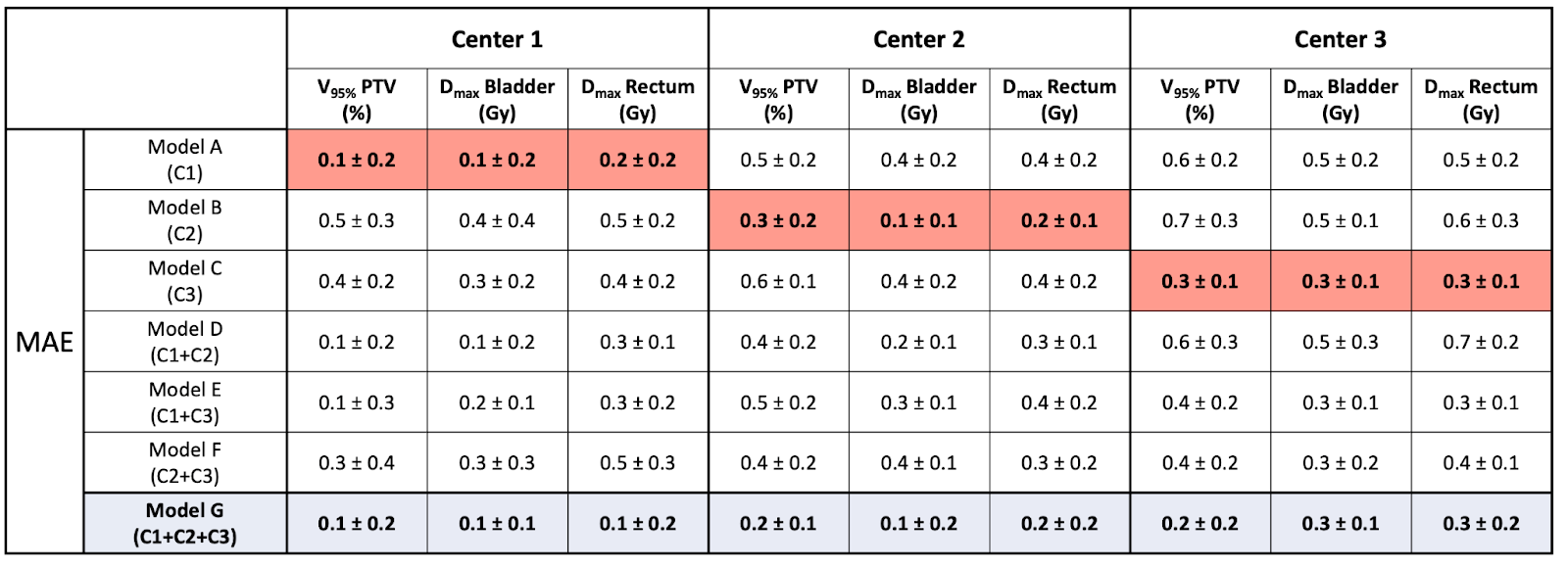


*Additional Table 1. Mean values of volume and dose difference calculated between CT and sCT for all the DVH indicators considered. Absolute dose values were reported in Gy for all the parameters investigated except for V_95%_ of PTV where the volume percentage difference was considered. The values are expressed as mean ± standard deviation.*

*Red cases indicate the monocentric models monocentric (Model A/Test C1, Model B/Test C2, Model C/Test C3) and blue cases represent the generic model (Model G/Test C1 or C2 or C3).*
